# Empyema Secondary to* Actinomyces meyeri* Treated Successfully with Ceftriaxone Followed by Doxycycline

**DOI:** 10.1155/2016/9627414

**Published:** 2016-09-26

**Authors:** Etienne Paris, Tonio Piscopo, Karen Cassar

**Affiliations:** ^1^Department of Medicine, Mater Dei Hospital, Msida, Malta; ^2^General Medicine and Infectious Diseases, Mater Dei Hospital, Msida, Malta; ^3^General and Acute Medicine, Mater Dei Hospital, Msida, Malta

## Abstract

Actinomycosis is a relatively rare infection caused by Gram-positive bacteria. We present the case of a 54-year-old, previously healthy, male patient with a history of severe penicillin allergy who developed severe pneumonia and empyema caused by* Actinomyces meyeri*. Presenting symptoms included productive cough, right upper quadrant pain, and chills and rigors. He required drainage of the empyema via tube and prolonged antibiotic treatment with intravenous ceftriaxone for 2 weeks followed by oral doxycycline for 6 months.

## 1. Introduction

Actinomycosis is a rare infectious disease caused by Gram-positive, non-spore-forming, predominantly anaerobic bacteria of the* Actinomyces* genus [1]. Most commonly, it presents as cervicofacial disease with the species* Actinomyces israelii* being most commonly implicated in humans [2]. However, it can virtually affect any body organ, with pulmonary involvement amounting to around 15% of cases [3]. Pulmonary actinomycosis presents with nonspecific symptoms and is a rather challenging diagnosis to make, being often misdiagnosed as tuberculosis, abscess, or lung malignancy. Penicillin remains the drug of choice in actinomycosis. In this report, we discuss a case of empyema caused by* Actinomyces meyeri* in a patient allergic to penicillin.

## 2. Case Presentation

A 54-year-old security officer presented with a 1-week history of intermittent abdominal pain, mainly in the right upper quadrant, occasionally radiating to the right loin, periumbilical area, and right lower chest. The pain was relieved with NSAIDs but worsened on inspiration and on sitting up. There was no relation to food or exertion. He also complained of chills and rigors and chronic productive cough.

He had no relevant past medical history other than a history of varicose vein avulsion a few years previously. However, significantly, he did report a history of penicillin allergy. He allegedly had an urticarial rash over the arms at 15 years of age when exposed to the drug. He subsequently never tried penicillin again. He was a smoker of 25 cigarettes daily and binged on alcohol during weekends.

On clinical examination, the patient was alert and fully oriented. He was apyrexial, with oxygen saturations of 94% in room air and a respiratory rate of 20 breaths per minute. Blood pressure and heart rate were within normal limits. Heart sounds were unremarkable. Respiratory examination revealed decreased air entry in the right base, with a stony dull percussion note. There were also few sparse expiratory wheezes. The abdomen was completely soft and nontender. Murphy's sign and renal punches were all negative. Multiple dental caries were evident, but otherwise there were no splinter haemorrhages, clubbing, or lymphadenopathy.

**Figure 1:**
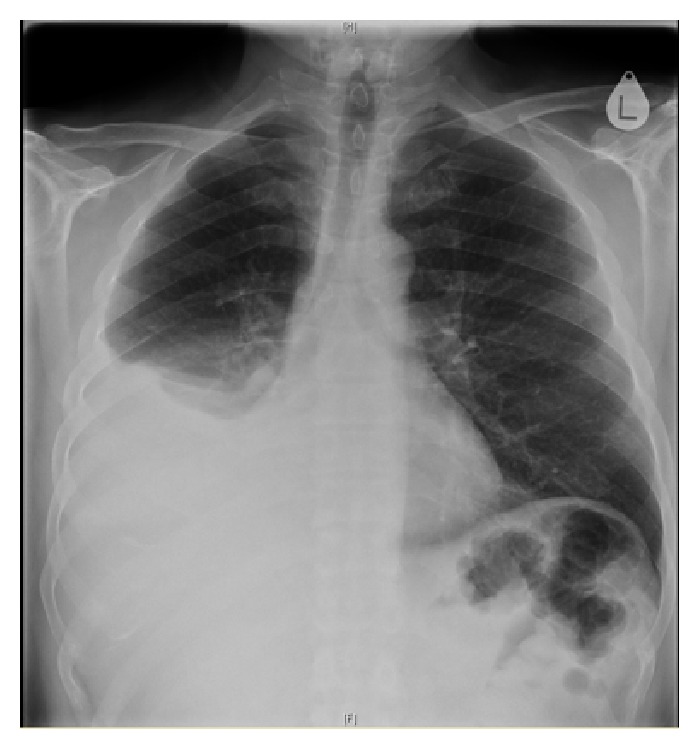
Large pleural effusion in the right hemithorax with probable collapse of right middle and lower lung lobes.

**Figure 2:**
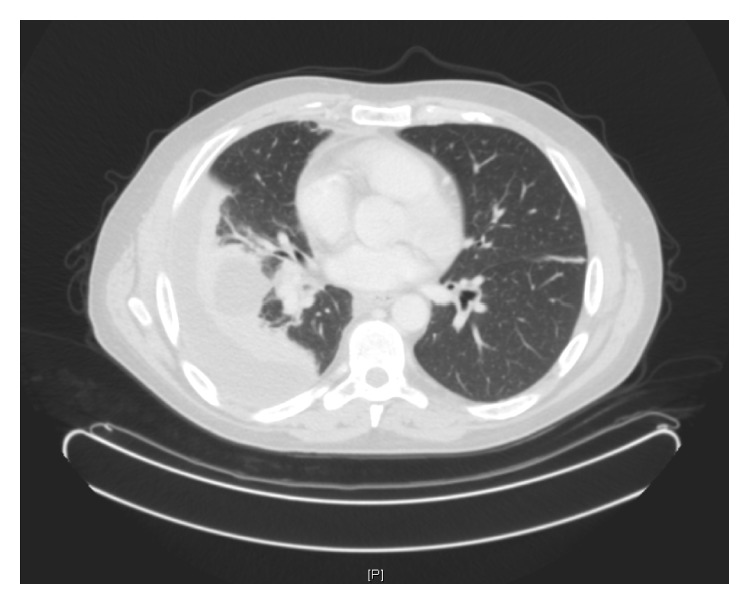
Computed tomography scan showing right-sided middle and lower lobe pulmonary consolidations with a moderately sized right-sided pleural effusion. There was no evidence of underlying malignancy.

Chest radiography confirmed a right-sided pleural effusion with probable collapse of the right middle and lower lung lobes (Figure 1). CT scan of the trunk revealed right-sided middle and lower lobe pulmonary consolidations with a moderate sized pleural effusion. There was no evidence of underlying malignancy (Figure 2). Blood tests revealed a leukocytosis with neutrophilia of 24.36 × 10^9^/L, an erythrocyte sedimentation rate of 79 mm/hour, C-reactive protein of 215 mg/L, and normal amylase level. Retroviral screen was negative.

15 mL of straw-coloured pleural fluid was aspirated under ultrasound guide and full aseptic technique. Fluid pH was 7.39, while fluid protein and LDH were increased at 57 g/L and 814 U/L, respectively. Cell count was within normal limits. Initial fluid cultures were negative. Intravenous levofloxacin and gentamicin were started after fluid aspiration.

Despite intravenous antibiotics, no improvement in inflammatory markers was observed over the next few days, and the patient also spiked a temperature of 38°C. The patient initially refused to undergo a therapeutic pleural tap but eventually consented to having one six days into his admission. This time, the pleural fluid, which was again an exudate, had a pH of 6, in keeping with an empyema. Gram-positive rods were detected in the pleural fluid. Till now, the patient had been on levofloxacin and gentamicin. Antibiotics were initially switched to meropenem and teicoplanin but later when culture of the pleural fluid revealed* Actinomyces meyeri* antibiotics were again changed to ceftriaxone 2 g daily. A chest drain was then inserted under sedation by the cardiothoracic surgeons.

**Figure 3:**
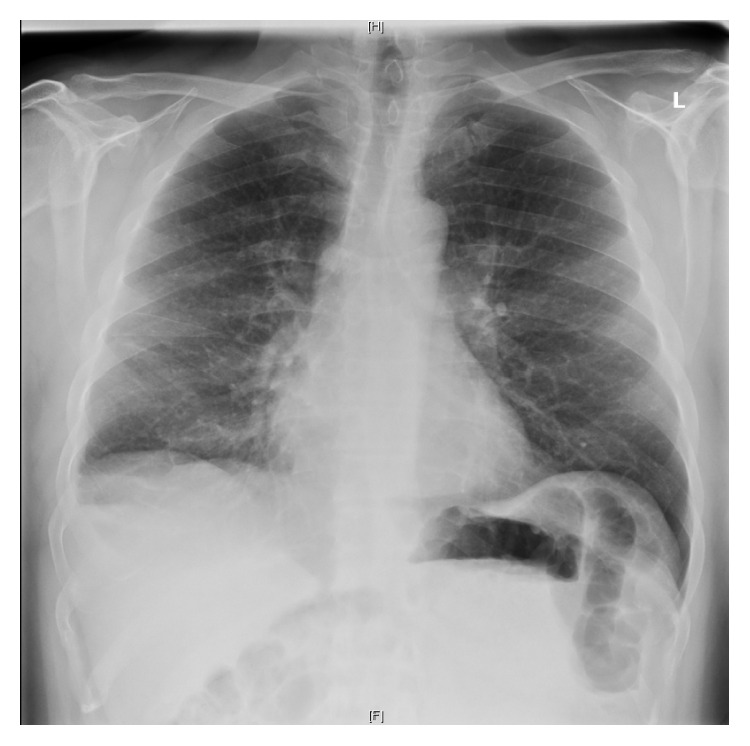
Chest radiography 6 months after diagnosis showing drastic improvement. The lungs were clear except for a small persistent right-sided pleural effusion. The cardiothoracic ratio and the mediastinal contours were unremarkable.

White cell count decreased from 27 × 10^9^/L to 14 × 10^9^/L, while C-reactive protein also decreased from 215 mg/L down to 100 mg/L. The patient's general condition, as well as blood tests and chest radiography, continued to improve. Ceftriaxone was continued for a total of 14 days and then switched to oral doxycycline 100 mg twice daily, which was continued for 6 months. He remained well, asymptomatic, and afebrile. The white cell count and C-reactive peptide remained within normal limits. Follow-up chest radiography showed a drastic improvement and almost complete resolution of the pleural effusion (Figure 3). The patient was also referred to a dentist for treatment of his dental caries.

## 3. Discussion

Members of the* Actinomyces* genus are Gram-positive bacteria, which grow best in anaerobic conditions. Colonies form fungus-like networks of hyphae, which led to the incorrect assumption in the past that this organism was a fungus and hence the name* Actinomyces* meaning “ray fungus” [1].

*Actinomyces* are commensals of the human oropharynx, gastrointestinal tract, and female reproductive system. Hence infection is almost always endogenous [3] and occurs when the mucosal barrier is disrupted because of, for example, inflammation, trauma, or surgery. In the case of* Actinomyces meyeri*, this usually affects the lungs and also has a tendency for haematogenous dissemination [4].

Pulmonary actinomycosis usually occurs in middle-aged and elderly males with a history of alcohol abuse and poor dental hygiene [4]. The commonest symptoms are dyspnoea, chest pain, and productive cough, and radiologic findings are nonspecific, making differentiation from pneumonia and malignancy very challenging. Isolation of the microorganism from specimens may also be difficult due to previous antibiotic use, growth of other organisms, and inappropriate laboratory methodology.

The first real breakthrough in the treatment of actinomycosis came in the 1930s with the use of sulphonamides until these were superseded by penicillin, which remains up to this day the treatment of choice. Conventional therapy for actinomycosis is high-dose intravenous penicillin at a dose of 18–24 million units for 2–6 weeks, followed by oral therapy with amoxicillin or penicillin V for 6–12 months [5]. In penicillin allergic patients, such as in the case we presented here, tetracyclines are a good alternative, while erythromycin can be used in pregnant, penicillin-sensitive women [1].

A study conducted in 2005 to assess the susceptibility of* Actinomyces* species to a variety of 12 antimicrobial agents recommended that *β*-lactam antibiotics are used as first-line treatment for* Actinomycosis*, combined with a *β*-lactamase inhibitor. Cephalosporins and tetracyclines, used in this case, were recommended as an alternative in penicillin allergic patients; however, this study claimed that the microbiological efficacy of these drugs is much less than that of aminopenicillins with or without a *β*-lactamase inhibitor [6]. Nevertheless, a number of reports claim complete clinical resolution of actinomycosis with ceftriaxone and doxycycline, used alone or together [7–9]. Metronidazole, fluoroquinolones, and aminoglycosides are not recommended as they are ineffective against actinomycetes [1, 6].

A review of English-language literature revealed only 6 case reports of confirmed* Actinomyces meyeri* empyema [4, 5, 9–12]. Five of the six patients described were males. In four cases, surgical thoracotomy was performed, while the other two required drainage of the empyema via chest drain as in our case. Duration of effective antibiotic therapy ranged from 4 to 13 months. In the present case, an early diagnosis was made and no dissemination was evident. Intravenous ceftriaxone followed by oral doxycycline was used due to penicillin allergy. The patient had no further temperature spikes and a review 6 months later found him well and asymptomatic.

## 4. Conclusion

Empyema due to* Actinomyces meyeri* is rare and is a difficult diagnosis to make. It should however be included in the differential diagnosis of nonresolving pneumonia and persistent pulmonary shadowing. The gold-standard treatment is high-dose penicillin but doxycycline has been shown to be effective and can be considered as a safe alternative, especially in cases of penicillin allergy [9]. In all cases described, including this one, successful management included a surgical drainage procedure [4, 5, 9–12].
